# Pragmatic reproducible research: improving the research process from raw data to results, bit by bit

**DOI:** 10.1172/JCI173741

**Published:** 2023-08-15

**Authors:** Luke V. Rasmussen, Eric W. Whitley, Leah J. Welty

**Affiliations:** Northwestern University Clinical and Translational Sciences Institute, Northwestern University, Chicago, Illinois, USA.

Reproducible research, defined here as the ability to recreate results given the same data, analytic code, and documentation (1), has received increasing attention in the past several decades (2–5). This definition of reproducibility has also been referred to as computational reproducibility (6). When it is infeasible to replicate a scientific study, such as a clinical trial, costly experiment, or longitudinal cohort study, reproducibility provides a minimum standard of scientific rigor (1). We note that for laboratory experiments, reproducibility is sometimes interpreted as setting up an experiment using the same methods and processes to yield the same conclusion. For the purposes of this Viewpoint, we will focus on reproducibility as the process of generating the same data analytic results given the raw data, and not on regenerating experimental data.

Reproducible research means that one could, as needed, repeatedly generate the same point estimates, confidence intervals, and P values from the raw data. Indeed, if one were to reanalyze the same data, using the same methods, and obtain different results each time, how would we ever know which conclusions to believe? Reproducibility cannot protect against errors (7) in raw data, nor can it guarantee the appropriate use of analytic methods, but it does provide a direct line of documentation from raw data to conclusions. Ultimately, such documentation may even help uncover errors in raw data or analytic steps.

Although there is widespread agreement that practicing reproducible research is a good idea, there is equally widespread variation in both how to practice reproducible research and what practicing reproducible research constitutes. The digitization of data and evolution of computer software for analysis and reporting of results means that tools and processes for practicing reproducibility are constantly changing. In the late 1980s, using a single code file to call other scripts to generate results constituted reproducibility (8); in the early 2000s, combining statistical code with document text in one file (typically using Sweave) was often considered synonymous with reproducibility in the statistics community. As of this writing, there are a vast array of software tools and platforms to support reproducibility. For example, there are tools that combine analytic code and its output with document text (e.g., R Markdown, Jupyter Notebook); provide platforms for sharing and running analytic code (e.g., Code Ocean, GitHub); provide open source project documentation and management (e.g., Open Science Framework); manage versions of data and code (e.g., Git, CVS, SVN); and create an executable snapshot of the environment used to run analytic code (e.g., Docker). Although these tools all support reproducibility, they do not define reproducibility.

To add complexity to the matter, there is often confusion about what constitutes conducting reproducible research. Practicing open science and replicating research are often conflated with reproducibility. Although definitions of open science vary, in general it requires that data and code be made broadly available (9). While it is true that open science may be reproducible, (a) it is not a requirement that data and code be made broadly available for the research itself to be reproducible, and (b) even if code and data are available, the results may not be reproducible. In our experience, fields in which data contain protected health information (PHI) or personally identifying information (PII) may be less likely to consider the sharing of data and code a requirement for reproducibility**.** Noting that definitions may vary across fields, we define replicating research as conducting an independent experiment or study to verify a result (7). However, replicating a finding does not imply the reproducibility of either the original or the replicated results.

Even after defining reproducibility and recognizing its importance, embracing reproducible research practices may seem daunting. Changing behavior is difficult, especially if not aligned with incentives of the research enterprise (10) or the individual scientist. Adopting new software that supports reproducibility may be time consuming and requires the development of additional technical skills. For large research teams, it may be difficult to convince the entire team to switch workflows and practices. In addition, some recommendations for reproducibility may seem dogmatic (i.e., insistence on a particular software or prescribed platform), which can reduce the motivation to try at all.

With these challenges in mind, we propose the idea of “pragmatic reproducible research.” Our approach is to frame reproducibility as a continuum of achievable practices (10, 11), dispelling misperceptions that it requires a complete shift in workflow or software tools before one can claim success. Our recommendations are not intended to provide specific instructions or particular guidance for how to conduct reproducible research, although we note that an increasing number of resources are available (e.g., 12–14). Our intention is to describe a pragmatic approach that will allow more scientists to move toward the goal.

We offer four primary recommendations, described in detail below: (a) reproducibility is about accounting for variation and change; (b) software tools can help but are not required; (c) an investigator can initiate these practices for their own benefit; and (d) reproducibility is not an all-or-nothing endeavor.

## Reproducibility is about accounting for variation and change

The goal of reproducible research is to obtain the same result given the same data, analytic code, and documentation. Irreproducible research occurs because something from the raw data to the conclusion changes. Research studies are increasingly large and complex — change can appear at many junctures, and perhaps not always where we expect it. For example, the people conducting the research can change over time — a biostatistician may leave, and the new biostatistician joining the team needs to know what decisions were made about what tests to perform or what parameters to use in a model. Data can also change — for example, discovering a mistake in a participant’s birth date. Updating the data set may result in two data sets, and it is important to know which one is the right one to use. By accounting (or controlling) for change, the scientist reduces unexplained variability in moving from raw data to conclusions.

Historically (and still today), a scientist would write their hypotheses, methods, and results in a laboratory notebook, allowing them to track the history of their experiments as they unfolded. They accounted for change by documenting what they did and what they observed, referring back to that documentation when it was time to reproduce the result. Therefore, a scientist can conduct reproducible research by focusing on improved record keeping (15). What has evolved is the complexity of keeping a record of more data and more complex methods for analysis. The good news is that software tools can help.

## Software tools can help but are not required

There are many useful tools that can aid a researcher in practicing reproducible research. Software can be used for data management, documenting study metadata, weaving analysis and results into manuscripts, and automation — to the point that it is possible to “press a button” and have all computational steps reproduced. For example, running a single Python script could access data from a Research Electronic Data Capture (REDCap) project, analyze the data, and generate a PDF version of a manuscript. The added benefit is that software can control for change (or variation) by automating processes that a human may perform differently each time. For example, having code run all steps of an analysis without human intervention can ensure that code is run in a set order and that a required parameter is not forgotten or mistyped. However, none of these tools is required for reproducible research.

A paper laboratory notebook may still have a place in today’s digital world, if that is what the scientist prefers to use and is willing to use. Additionally, electronic documents (e.g., Notepad or Microsoft Word) can be used to capture notes and allow for indexing and searching at a later date. While scientists should follow practices and conventions for reproducibility at their own laboratory or institution, they can and should consider how additional tools or processes may assist them in their research workflows. However, scientists should never feel that they must adopt a specific tool in order to be doing reproducible research “right.”

## An investigator can initiate reproducible research practices for their own benefit

There are many reasons why someone would want to conduct reproducible research. These can be altruistic: reproducible research adds rigor and transparency to the research process and may help other scientists fully understand the methods or allow them to reproduce and replicate the work. While these are certainly valid and beneficial reasons, it is important to recognize that reproducible research also requires an investment of time and effort by a scientist.

Early work promoting reproducible research, especially in the geophysical sciences in the 1980s, was in part motivated by the fact that researchers had “difficulty reproducing their own computations without considerable agony” (8). Pragmatically, the individual scientist may benefit from conducting reproducible research even more than their colleagues do. We often describe conducting reproducible research as a benefit to “our future self.” That is to say, very rarely does the effort put into additional documentation or automation help the individual today; instead, it provides a future benefit when summarizing, explaining, or justifying results. For example, when writing the manuscript, it is critical to list the covariates used for adjusting a regression coefficient. Having that documentation as part of the research workflow will save time double-checking estimates.

With research involving larger investigative teams, another consideration is whether everyone on the team must be practicing reproducible research in order to reap the benefits. Although it is certainly advantageous if the entire team appreciates the value of reproducible research, it may not be feasible to convince everyone to take the leap. Even if a single scientist on the team is focused on making their part of the research reproducible, such an effort benefits them, the larger team, and most likely the research itself. A scientist should not discard the idea of performing reproducible research for their part of the research workflow, as it may in fact serve as a motivational example for others on the team. In contrast, larger teams may, by necessity, adopt formal or written processes for where to store data, how to name files, and who has read/write access, and these practices benefit the individual scientist as well. In addition, smaller teams who practice reproducibility may find it easier to expand their research portfolios. For example, if postdoctoral fellows store data and generate code with an eye to reproducibility, the documentation will aid the transition of papers and projects from one postdoctoral fellow to the next.

## Reproducibility is not an all-or-nothing endeavor

Reproducible research can be thought of as a continuum; scientists may be conducting reproducible research at any point along the way. This continuum flows across different axes. For example, there are different phases of research — from data collection, data cleaning, data analysis, preparation of figures and tables, and drafting a manuscript, to finalizing the publication proofs. Reproducible research practices can play a role in any and all of the phases, and the scientist can consider which phase(s) they want to start with. For example, if a scientist is comfortable writing R code to automate data cleaning and data analysis, they may choose to do that first, and over time learn more about using R Markdown to generate their manuscript.

Another axis is perhaps best framed as the technical maturity of the process (although it is worth reiterating that a less mature process is not necessarily a bad thing!). As an example, to document an analysis using bioinformatics tools from the command line, a scientist may begin by keeping a log of the steps and commands in an electronic document. As they build the habit of documenting the process, they may consider transitioning to a specific tool that integrates command line and documentation, such as a Jupyter Notebook or a makefile.

## Conclusion

Recognizing a problem means it is possible to address it. With increased awareness of the benefits of reproducible research, general concepts and formal methods are being added to existing curricula and other training courses, and a wide range of computational tools are available to support reproducible research practices across the span of the research lifecycle. Noting the importance of reproducible research and the benefits it offers, we have reframed the goals and activities of reproducible research in a more pragmatic light. It is important to dispel myths or misperceptions about the complexity of reproducible research to reduce barriers to adopting practices that support reproducibility. Perhaps more easily summarized, the key idea behind a pragmatic view of reproducible research is this: just get started.

## Version 1 08/15/2023

Electronic publication
